# Comparison of cytokine profile of IFN-γ, IL-5 and IL-10 in cutaneous leishmaniasis using PBMC *vs.* whole blood

**Published:** 2019-10

**Authors:** Akram Miramin Mohammadi, Amir Javadi, Alireza Firooz, Ali Khamesipour

**Affiliations:** 1 Center for Research and Training in Skin Diseases and Leprosy, Tehran University of Medical Sciences, Tehran, Iran; 2 Department of Social Medicines, Qazvin University of Medical Sciences, Qazvin, Iran

**Keywords:** Leishmaniasis immune response, Peripheral blood mononuclear cells, Whole blood

## Abstract

**Background and Objectives:**

The surrogate marker (s) of cure and protection in intracellular pathogens is not yet well defined. The aim of this study was to compare the cytokine profile using whole blood cells (WBC) vs. peripheral blood mononuclear cells (PBMC) in healthy and cutaneous leishmaniasis (CL) volunteers.

**Materials and Methods:**

In this study, WBC and PBMC of the volunteers with history of CL (HCL), Active lesion (ACL) and healthy volunteers were collected. The WBC and PBMC were cultured and stimulated with either PHA or soluble *Leishmania* antigens (SLA), after 72 hours, the supernatants were collected and the levels of IFN-γ, IL-5 and IL-10 were titrated using ELISA method.

**Results:**

The mean ± SD of cytokines using WBC and PBMC in cutaneous leishmaniasis volunteers stimulated with phytohemagglutin (PHA) or SLA are as follow, PHA, IFN-γ=2295±995 vs. 2339±1115, IL-10=853±309 vs. 1330±966, and IL-5=299±136 vs. 352+156, SLA, IFN-γ, 931±824 vs. 825±532, IL-10, 233±78 vs. 408±381, and IL-5, 185±59 vs. 217±76, respectively. There was no significant difference between the IFN-γ, IL-5 and IL-10 levels using WBC vs. PBMC. There was a strong correlation between the cytokine profiles using WBC and PBMC in cutaneous leishmaniasis volunteers.

**Conclusion:**

There was no significant difference between IFN-γ, IL-10, IL-5 levels in whole blood and PBMC of volunteers with active lesion or history of CL. Whole-blood culture which is easier, cheaper and more convenient could be used instead of PBMC to evaluate the cytokine profile in field conditions.

## INTRODUCTION

Leishmaniasis is a neglected tropical disease caused by different species of *Leishmania*, the disease is reported from over 100 countries in tropical and subtropical parts of the world. Leishmaniasis is endemic in the poorest parts of developing countries usually with a limited infrastructure. Depending upon the type of host immune response and the species of the causative agent, *Leishmania* infection induces a range of outcomes, from asymptomatic to diverse clinical manifestations including cutaneous leishmaniasis (CL) which is a self-healing skin lesion and the most common form of the disease to a systemic life-threatening form of visceral leishmaniasis. Development of vaccine against leishmaniasis seems to be the most effective strategy to control the disease. Efforts to develop a vaccine against leishmaniasis has not been successful yet, the main difficulty in development of a vaccine against the disease is limited information about the mechanism (s) of protection. The surrogate marker (s) of protection in intracellular infectious diseases is yet not well defined, identification of the surrogate marker (s) of protection in disease such as leishmaniasis facilitates vaccine development and possibly improves treatment strategies (1, 2).

In experimental murine model of leishmaniasis, the outcome of *Leishmania* infection mainly depends upon the type of generated immune response; in resistant strains of mice, upon infection with *L. major* a Th1 response is induced and as a result a disease similar to human CL develops and when the lesion cured, the animals are protected against further infection, in contrary in susceptible BALB/c mice, infection with *L. major* induces a Th2 type of immune response which accompanies a severe systemic disease and eventually every infected animal succumbed to the disease (3–5). Although in the murine model of *L. major* infection generation of Th1 response accompanies with the cure and protection against further infection, but recent evidences showed that not only in human but also in murine model of leishmaniasis, only Th1/Th2 response cannot explain the whole story of cure and protection. The surrogate marker (s) of protection in leishmaniasis is not well defined and needs to be investigated. In leishmaniasis endemic areas usually, infrastructure is poor and not enough to study human immune responses with enough sample size to reach to a conclusion, isolation of peripheral mononuclear cells (PBMC) make it more difficult due to lack of the laboratory in vicinity of endemic areas, the possibility of using whole blood instead of PBMC in endemic areas makes it much easier to evaluate the immune responses and possibly define the surrogate marker (s) of protection. Evaluation of immune response using whole blood (WBC) is much more convenient than isolation of peripheral blood mononuclear cells (PBMC) (19).

The aim of this study is to compare the Th1/Th2 cytokine profiles using whole blood *vs.* PBMC.

## MATERIALS AND METHODS

### Ethical consideration and participants

The proposal of the study was approved by the institutional ethical committees of Tehran University of Medical Sciences. The potential candidate was interviewed, and the objective and the procedures of the study were explained to her/him, the one who was willing to participate, donate blood samples and sign an informed consent was recruited.

### Participants

The patients with active lesion suspected of CL were referred to leishmaniasis clinic of Center for Research and Training in Skin Diseases and Leprosy (CRTSDL), interviewed and then visited by a physician and were physically examined. Skin samples were collected from every patient’s lesion for direct smear, culture and PCR. When parasites were found in the lesion the patient was requested to read the informed consent and decide to participate in the study. Diagnosis, treatment, and management of the lesions even of those who refused to participate in the study were completed free of charge. Individuals (18 to 60 years old) with history of CL (HCL) who previously were treated at leishmaniasis clinic of CRTSDL also were recruited if agreed to donate blood samples and sign an informed consent.

Every involved volunteer [(HCL (n=5), ACL (n=5)] was the same as above, and the *Leishmania* species was identified using PCR. The healthy volunteers (n=10) were recruited from the medical staffs of the center who were willing to participate, donate blood samples and sign an informed consent.

### Parasites and antigens

*L. major* (MRHO/IR/75/ER) was used in this study, this is the same isolate which was used in mass leishmanization, preparation of leishmanin and first generation experimental vaccines (6). To maintain pathogenicity of *Leishmania*, the isolates were inoculated into BALB/c mouse, the parasites were isolated from the spleen of the infected mouse, cultured in NNN media and then sub-cultured in complete RPMI media supplemented with 10% FCS and 1% penicillin/streptomycin (7).

### Preparation process of soluble *Leishmania* antigen (SLA)

SLA was prepared using the protocol developed by Scott P. et al. (8) with minor modifications, briefly, the parasites were harvested at stationary phase and washed 4 times using HEPES-sucrose buffer (10 mM, 10% w/v, pH=7.4). Then, the number of promastigotes was adjusted to 1.2×10^9^/ml in a buffer solution containing 50 μl/ml enzyme inhibitor cocktail, (Sigma, St. Louis, USA). Then, the parasites were lysed using freeze-thaw method followed by probe sonication in an ice bath. The supernatant of centrifuged lysate parasites was collected, dialyzed against buffer solution, sterilized using a 0.22 μm membrane and stored at −70°C until use. The protein concentration of the preparation was determined using the bicinchoninic acid (BCA) protein assay method (Thermo Scientific, USA).

### Blood sample collection

Twenty ml heparinized blood sample was collected from each volunteer. The blood sample was divided into two parts, one part was directly used to culture as whole blood, and the second part was used to isolate PBMC, the blood sample was diluted 1:1 using RPMI, and overlaid gradually with Ficoll-Hypaque (30–40% of blood volume) in 50 ml disposable centrifuge tubes. Peripheral blood mononuclear cells (PBMC) were isolated from the peripheral blood, washed three times using PBS and resuspended in RPMI containing 10% heat inactivated fetal calf serum and 100 μ/ml penicillin and 100 μ/ml streptomycin. The cell number was counted and PBMC was adjusted to 2×10^6^ cells per 1 ml of complete RPMI and then 200 μl volume containing 2×10^5^ cells were added to each well (9–11).

### Cell culture of whole blood

Four hundred fifty microliters of diluted blood (1:1 blood to RPMI) was added to each tube of 50 μL media and stimulated with either PHA (5 μg/ml), SLA (10 μg/ml), or with no stimulation in secure cap, the tube was tabbed 8–10 times to mix well, and 450 μL of blood was added to each tube. The tubes were placed in upright position and incubated at 37°C with 5% CO_2_ for 72 hours. Then, 500 μL of whole blood (WBC) solution was added to the 1.1 mL serum separator tube. The tubes were centrifuged at 10,000 rpm for 10 minutes at room temperature. The supernatant was transferred into a cryovial and kept at −70°C until usage (4, 12).

### PBMC culture

The cell number was adjusted to a final concentration of 1×10^6^ PBMC/ml, the cells were cultured in triplicates in U-bottom 96 well culture plates (2×10^5^ cells in 200 μl volume/well) and incubated for 72 h at 37°C with 5% CO_2_, the PBMC were stimulated with either PHA (5 μg/ml), SLA (10 μg/ml), or unstimulated as control. After 72 hours of incubation, 150 μl of supernatant was collected carefully from each well, and the triplicates were pooled and kept at −70°C until usage.

### Cytokine assay

IFN-γ, IL-10 and IL-5 levels of the supernatants were titrated using ELISA according to the manufacturers’ instructions (eBiosciences, USA). The results are expressed in picogram/ml (mean ± SD) of the triplicate experiments.

### Statistical method

Continuous variables are described as mean ± SD, categorical and ordinal variables are presented as proportions. The findings are presented as mean and 95% confidence intervals (CI). Comparison of the mean for variables with normal distribution between the two groups was done using T-test and the data with more than two groups was analyzed using ANOVA test. Comparison of median for variables with non-parametric distribution between the two groups was done by using Wilcoxon Signed Rank Test and the data with more than two groups was done using Friedman test. To compare the proportions, Pearson chi-square test or Fisher’s exact was used. Statistical significance was considered when probability value was less than 0.05. The MATLAB Version 2014 and SPSS Version 16 (SPSS Inc., Chicago, IL, USA) software was used for statistical computation.

## RESULTS

**Fig. 1:**
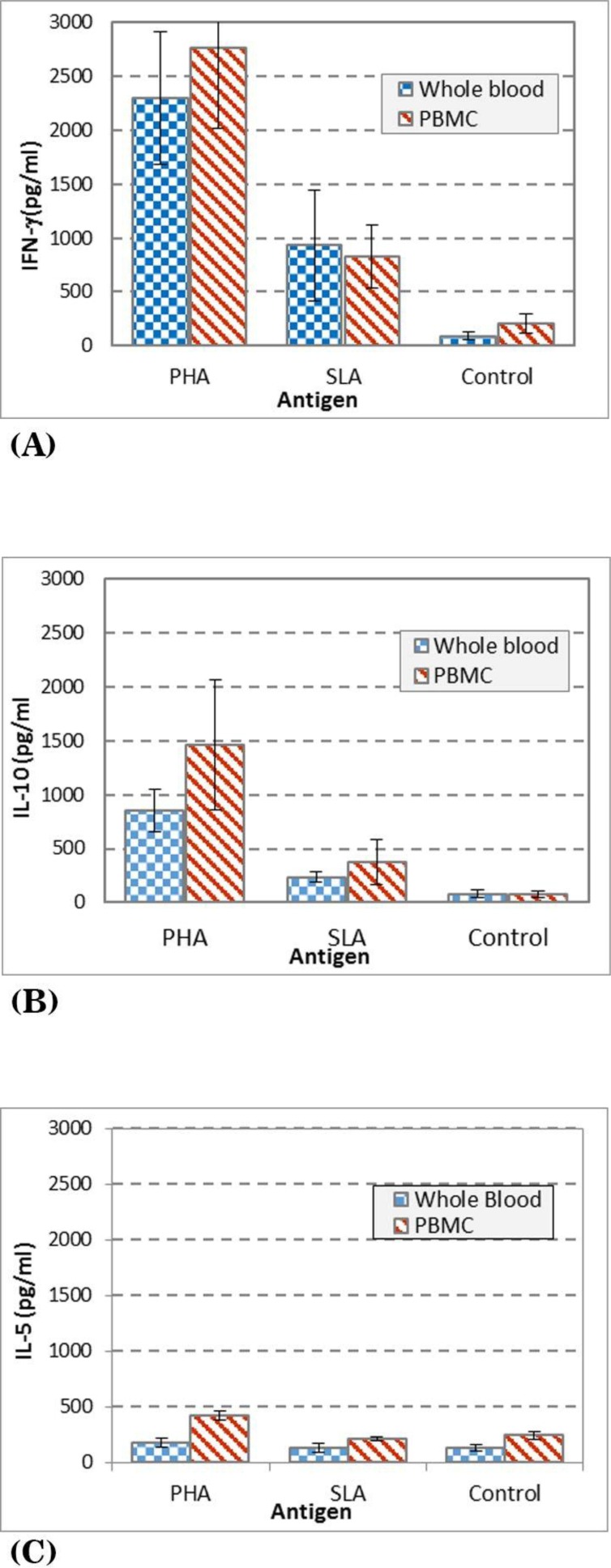
Cytokine levels in culture supernatants of whole blood and PBMC of volunteers with active CL lesion or history of CL stimulated with either PHA, SLA (soluble *Leishmania* antigens), or no stimulation as control. (A) IFN-γ level in culture (B) IL-10 level in culture (C) IL-5 level in culture

The background information of the volunteers including gender, age and duration of lesion and species of *Leishmania* are presented in Table 1. The results of IFN-γ levels of volunteers with history of CL and patients with active CL lesion are presented in Fig. 1A, as it is shown the mean + SD, in supernatants of the whole blood vs. PBMC stimulated with PHA is 2295 ± 995 vs. 2339 ± 1115, SLA is 931 ± 824 vs. 825 ± 532, respectively. The results of IL-10 levels are presented in Fig. 1B as it is shown the mean ± SD levels of IL-10 in supernatants of whole blood vs. PBMC stimulated with PHA is 853 ± 309 vs. 1330 ± 966, SLA is 233 ± 78 vs. 408 ± 381, respectively. The results of IL-5 levels are presented in Fig. 1C as it is shown the mean ± SD levels of IL-5 in supernatants of whole blood vs. PBMC stimulated with PHA is 299 ± 136 vs. 352 ± 156, and SLA is 185 ± 59 vs. 217 ± 76, respectively.

**Table 1:** Characteristic of the volunteers

	**Age**	**Sex**		**Average of duration of lesion per month**	**Species of *Leishmania***	
	
		**Male**	**Female**		***L. major***	***L. tropica***
HCL	34.8	3	2	6	4	1
ACL	38.4	3	2	2	4	1
Healthy	35.5	6	4	-	-	-

The mean ± SD of IFN-γ production in whole blood of volunteers with active CL lesion (s) and history of CL stimulated with SLA was significantly (p<0.005) higher than the culture control with no stimulation (mean ± SD, 931 ± 824 vs. 136 ± 70). The mean ± SD IFN-γ production in PBMC of volunteers with active CL lesion (s) or history of CL stimulated with SLA was significantly (P<0.001) higher than the culture control with no stimulation (mean ± SD, 825 ± 532 vs. 203 ± 161) (Fig. 1A).

Fig. 1B, shows the mean ± SD of IL-10 production in WBC of volunteers with active CL lesion (s) or history of CL stimulated with SLA which is significantly (P=0.005) higher than the control culture with no stimulation (mean ± SD, 233 ± 78 vs. 78 ± 54), and the mean ± SD of IL-10 production in PBMC of volunteers stimulated with SLA was significantly (P<0.005) higher than the control culture with no stimulation (mean ± SD, 408 ± 381 vs. 76 ± 51).

Fig. 1C shows that IL-5 production in WBC and PBMC of volunteers with active CL lesion (s) or history of CL stimulated with SLA is similar as the control culture with no stimulation (P>0.05).

**Fig. 2:**
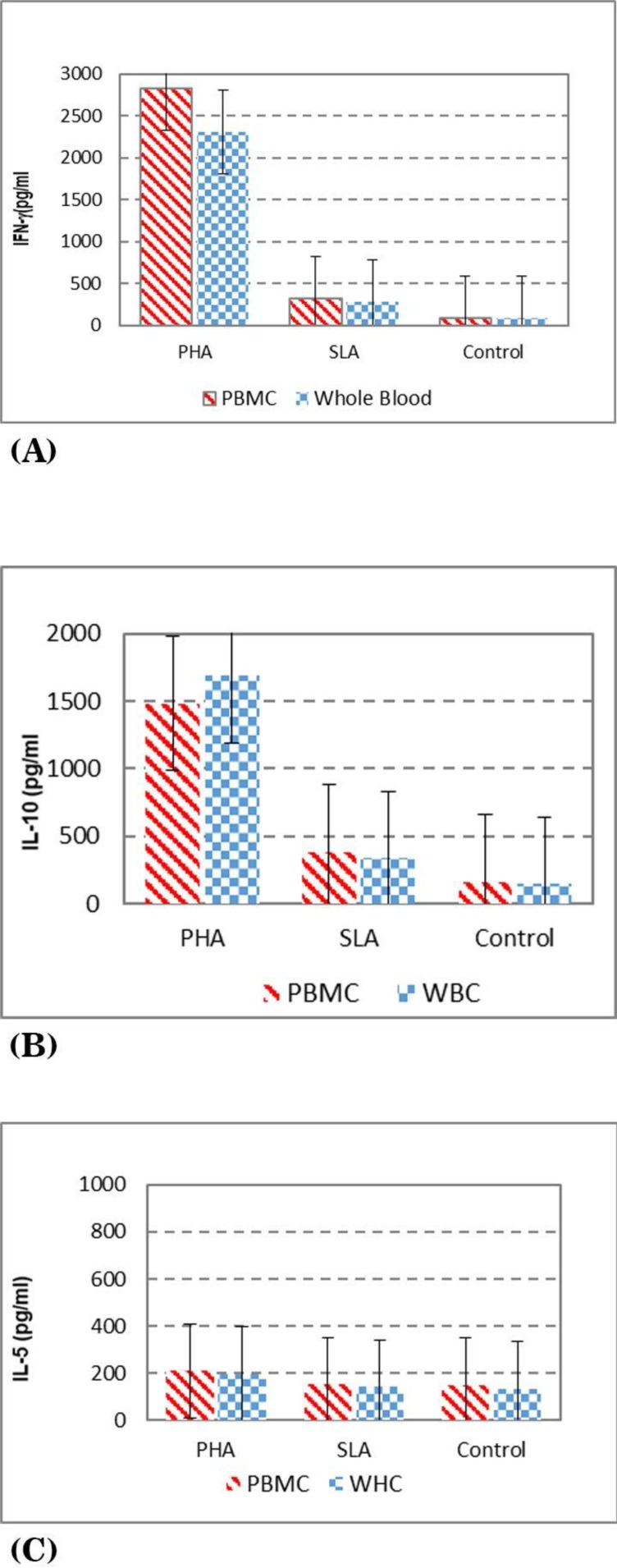
Cytokine levels in culture supernatants of PBMC and whole blood of healthy volunteers stimulated with either PHA, SLA (soluble *Leishmania* antigens) or no stimulation as control. A) IFN-γ level in culture (B) IL-10 level in culture (C) IL-5 level in culture

The results of IFN-γ levels in healthy volunteers are presented in Fig. 2A, as it is shown the mean + SD, in the supernatants of whole blood vs. PBMC stimulated with PHA is 2307 ± 672, 2821 ± 742, SLA is 281 ± 206, 314 ± 237, respectively. The results of IL-10 levels are presented in Fig. 2B as it is shown the mean ± SD levels of IL-10 in supernatants of whole blood vs. PBMC stimulated with PHA is 1689 ± 759, 1483 ± 734, SLA is 334 ± 221, 378 ± 275, respectively. The results of IL-5 levels are presented in Fig. 2C as it is shown the mean ± SD levels of IL-5 in supernatants of whole blood vs. PBMC stimulated with PHA is 196 ± 87, 209 ± 5 and SLA is 140 ± 33, 152 ± 24, respectively.

The mean ± SD of IFN-γ production in whole blood of volunteers with active CL lesion (s) or volunteers with history of CL stimulated with SLA was significantly (p<0.05) higher than the healthy volunteers (mean ± SD, 931 + 824 vs. 281 ± 206). The mean ± SD IFN-γ production in PBMC of volunteers with active CL lesion (s) or volunteers with history of CL stimulated with SLA was significantly (P<0.05) higher than the healthy volunteers (mean ± SD 825.8 + 532.1, vs. 314 ± 237) (Fig. 2A).

Fig. (2B), shows the mean ± SD of IL-10 production in WBC of volunteers with active CL lesion (s) or volunteers with history of CL stimulated with SLA was not significantly different from those of healthy volunteers (mean ± SD vs. 233.2 + 78.1, 334 ± 221), and the mean ± SD of IL-10 production in PBMC of volunteers with active CL lesion (s) or volunteers with history of CL stimulated with SLA was not significantly different from healthy volunteers (mean ± SD 378.1 + 380.9 vs. 378 ± 275).

Fig. 2C shows the IL-5 production in WBC of volunteers with active CL lesion (s) or volunteers with history of CL stimulated with SLA is similar to the ones from the healthy volunteers (mean ± SD 171.7 + vs. 56.0., 140 ± 33), but there was a significant (P<0.05) difference in IL-5 production in PBMC of volunteers with active CL lesion (s) or volunteers with history of CL stimulated with SLA vs. the ones from healthy volunteers (mean ± SD 217.6 + 76.6 vs. 152.4 ± 24.6).

There was no significant difference between IFN-γ, IL-5 and IL-10 levels using whole blood vs. PBMC in healthy volunteers.

**Fig. 3:**
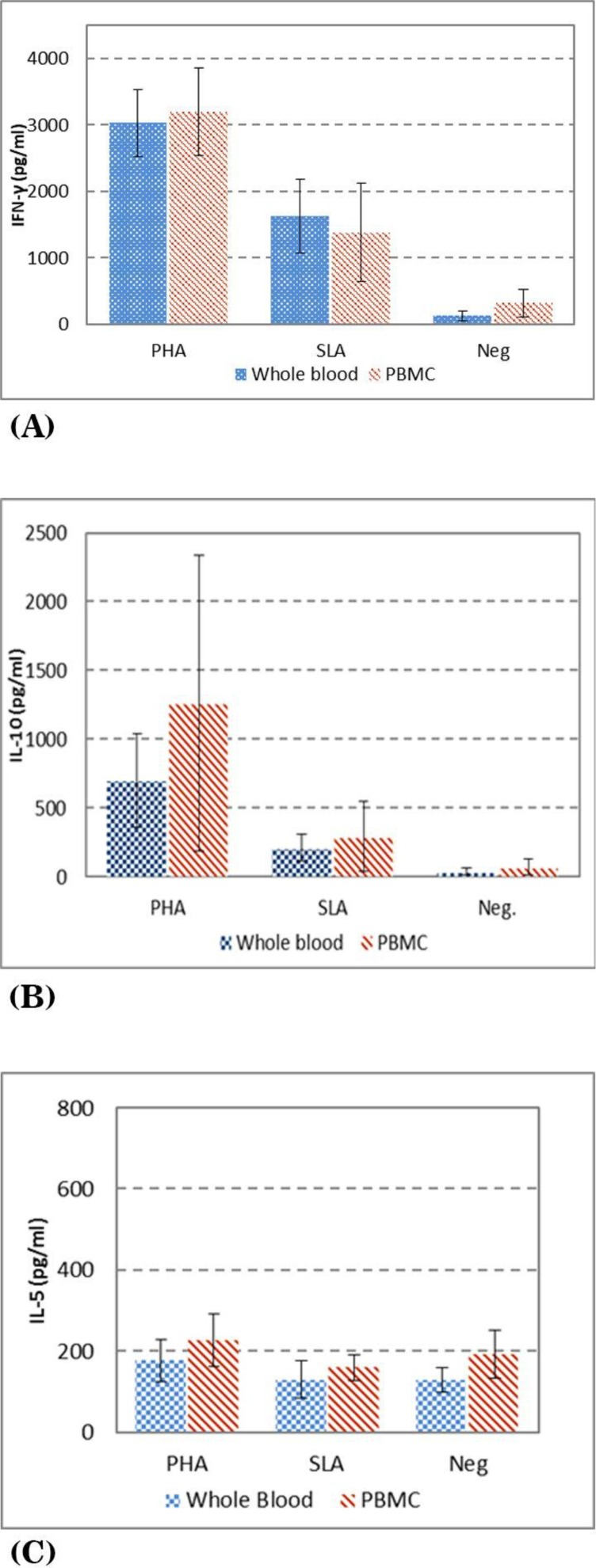
Cytokine levels in culture supernatants of PBMC and whole blood of history of CL volunteers stimulated with either PHA, SLA (soluble *Leishmania* antigens) or no stimulation as control A) IFN-γ in culture B) IL-10 level in culture C) IL-5 level in culture

The results of IFN-γ levels of volunteers with history of CL are presented in Fig. (3A), as it is shown the mean + SD, in supernatants of the whole blood vs. PBMC stimulated with PHA is 3032 ± 507. vs. 3195.8 ± 655.3, SLA is 1629.6 ± 552.4 vs. 1375.6 ± 784.3, respectively. The results of IL-10 levels are presented in Fig. (3B) as it is shown the mean ± SD levels of IL-10 in supernatants of whole blood vs. PBMC stimulated with PHA is 699.3 ± 344.4 vs. 882.6 ± 540.6, SLA is 210.4 ± 98.8 vs. 355.0 ± 276.1, respectively. The results of IL-5 levels are presented in Fig. (3C) as it is shown the mean ± SD levels of IL-5 in supernatants of whole blood vs. PBMC stimulated with PHA is 176.7 ± 50.9 vs. 226.3 ± 64.0, and SLA is 129.4 ± 46.1 vs. 158.0 ± 31.3, respectively.

**Fig. 4:**
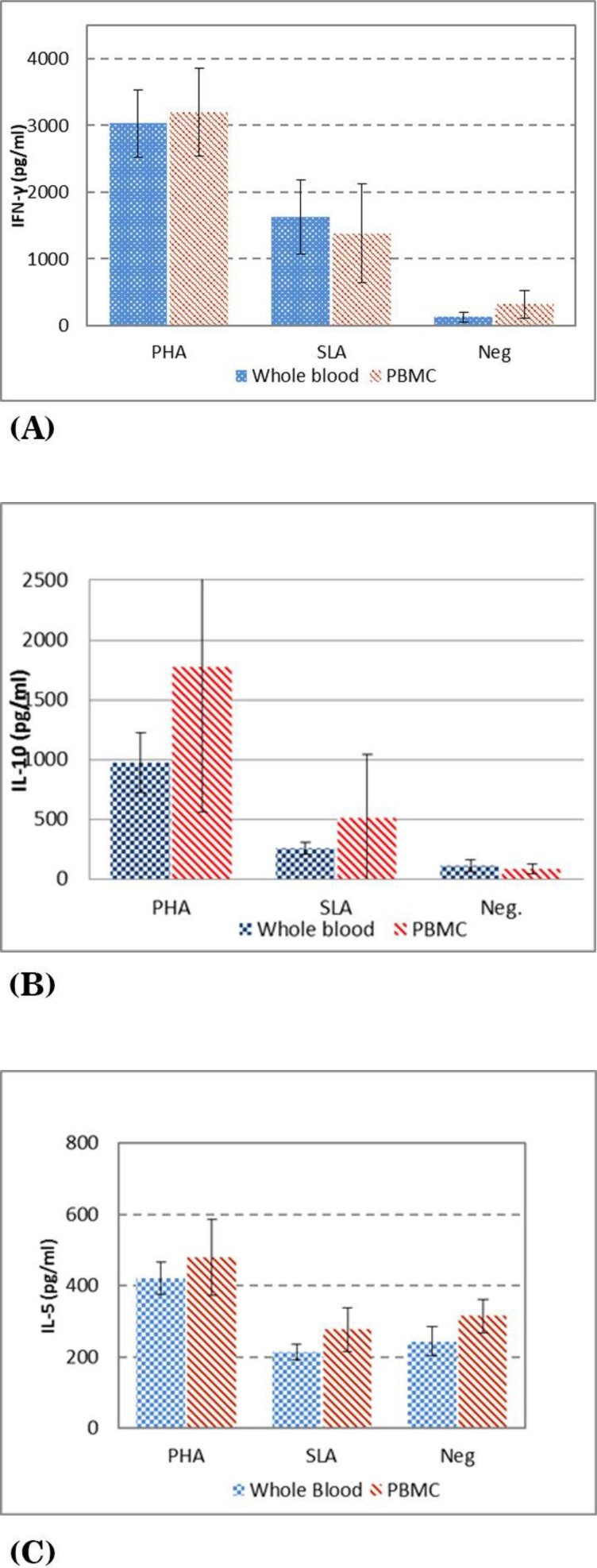
Cytokine levels in culture supernatants of PBMC and whole blood of Active of CL volunteers stimulated with either PHA, SLA (soluble *Leishmania* antigens) or no stimulation as control A) IFN-γ in culture B) IL-10 level in culture C) IL-5 level in culture

The results of IFN-γ levels of volunteers with active of CL are presented in Fig. (4A), as it is shown the mean + SD, in supernatants of the whole blood vs. PBMC stimulated with PHA is 1558.6 ± 784. vs. 1458.0 ± 730.5, SLA is 232.6 ± 73.8 vs. 275.2 ± 90.1, respectively. The results of IL-10 levels are presented in Fig. (4B) as it is shown the mean ± SD levels of IL-10 in supernatants of whole blood vs. PBMC stimulated with PHA is 976.0.3 ± 250.7 vs.1770.4 ± 1209.9, SLA is 256.0.4 ± 51.8 vs. 510.0 ± 534.9, respectively. The results of IL-5 levels are presented in Fig. (4C) as it is shown the mean ± SD levels of IL-5 in supernatants of whole blood vs. PBMC stimulated with PHA is 421.4 ± 44.8 vs. 477.8 ± 106.9, and SLA is 214.0.0 ± 21.8 vs. 276.2 ± 60.4.1, respectively.

There was no significant difference between IFN-γ, IL-5 and IL-10 levels using whole blood vs. PBMC in volunteers with history of CL.

There was no significant difference between IFN-γ, IL-5 and IL-10 levels using whole blood vs. PBMC in volunteers with active CL lesion.

There was no significant difference between IFN-γ, IL-5 and IL-10 levels using whole blood vs. PBMC in healthy volunteers.

## DISCUSSION

Although, the vaccine was developed for many extracellular infectious diseases but yet there is no vaccine available against many intracellular parasites (13). Leishmaniasis caused by different *Leishmania* species, the disease is endemic in many parts of the world, control management of the disease and treatment is not an easy task especially with some forms of leishmaniasis (2). There is no vaccine available against any form of leishmaniasis and many other diseases caused by intracellular parasites. The surrogate marker (s) of protection in extracellular infections is well known and as such the status of immune responses against specific extracellular pathogens can be easily assessed and as such estimation of protection rate and vaccine efficacy is feasible, but in the diseases which caused by intracellular pathogens, the surrogate marker (s) of protection is not well defined and protection rate could be only checked through clinical trials which is time-consuming and expensive. Identification of the marker (s) of protection is an essential step for development of vaccine against intracellular parasites prior to clinical trials, one of the reasons that there is no vaccine available against any intracellular parasites including leishmaniasis is lack of information about the surrogate marker (s) of protection. The easier process of immune response evaluation is the more data can be generated toward identification of surrogate marker (s) of protection. PBMC is routinely used to evaluate human immune responses in many studies especially to test immune responses in human leishmaniasis (10, 14–16), but the assay of isolation of PBMC is time-consuming, expensive and needs infrastructure which might not be available in endemic areas (17), moreover the process of isolation of PBMC might damage or change some of the immune cells’ functions. Using whole blood culture offers an appropriate condition and easy procedure to study leukocyte cytokine profiles instead of using PBMC (18). In a study using WBC and PBMC of healthy volunteers, cell proliferation and secreted IFN-γ against the purified recombinant antigen of *M. tuberculosis* and PHA were compared. The results showed a positive correlation between each assay and no significant difference was seen. In another study which was completed in TB patients, 17 cytokines were evaluated against *M. tuberculosis* antigens using whole blood vs. PBMC (2×10^5^ cells/ml), the results showed no difference between the cytokine titration of using WBC and PBMC (17). Using whole blood instead of PBMC offers an opportunity to collect the blood samples in the field and start culture procedures at the site of blood collection, the culture then can be transferred to the laboratory, in addition when using WBC less blood is needed compared to PBMC which is a great advantage in the case of vulnerable patients and children, more tests could be run using the same amount of blood (19). Using whole blood is closer to physiological condition, isolation of PBMC is most commonly done using density gradient centrifugation method which might change the immune cells proportion and function and also eliminates some cytokines, as well as cells such as granulocytes and platelets. It is shown that isolation of PBMC damages the cells and facilitate apoptosis (18). In one study the cytokine production by human whole-blood was compared with cytokine production by isolated monocytes and isolated PBMC. The authors found that the correlation between cytokine production was stronger between WBC and monocyte than between WBC and PBMC (20). Disadvantages of using whole blood are that the number of different cell types when using whole blood is not clear but using PBMC the exact number of cells which contribute to immune response generation is known. Evaluation of a specific type of immune cells is not possible using whole blood.

In this study, the whole blood was used to evaluate the immune responses in cutaneous leishmaniasis, WBC and PBMC from the same subjects were cultured and stimulated with either mitogen PHA which is a polyclonal stimulator or specific soluble *Leishmania* antigen (SLA), then the supernatants of the cultures were collected and used to titrate cytokines of IFN-γ, IL-5, and IL-10. The cytokines which were titrated from the WBC were compared with the cytokines which were titrated from the PBMC cultures.

The results showed that there was no significant difference between the cytokine titers of IFN-γ, IL-10 and IL-5 using the whole blood and the PBMC stimulated by either PHA (P=0.87, P=0.17, P=0.053), or SLA (P=0.247, P=0.149, P=0.056), respectively. The data which was generated using WBC was not significantly different from the data generated using PBMC. As it is shown by the current study and the other studies there is no significant difference in evaluation of immune responses using WBC and PBMC and there is a positive correlation between the cytokine titers using WBC and PBMC (21, 22). In this study, there was a correlation between the cytokine production using the whole blood and PBMC when evaluating the immune responses against polyclonal antigens PHA (correlation coefficient=0.8, P<0.001) and specific *Leishmania* antigens (correlation coefficient=0.57, P=0.001).

Studies on purified PBMC vs. whole blood have shown that using purified cell is less accurate in particular since the diverse cell subsets are not similar to whole blood, the purification might damage the cells. It is shown that PBMC cultures have had lower cell viability than whole blood, probably due to PBMC separation procedures (18). Overall using the whole blood is more practical in endemic areas with a limited infrastructure.
